# Advocate: A Tribute to Karen A. Goraleski

**DOI:** 10.4269/ajtmh.25-0090

**Published:** 2025-03-05

**Authors:** Chandy C. John, Patricia F. Walker, Daniel G. Bausch

**Affiliations:** ^1^ Indiana University, Bloomington, Indiana;; ^2^ University of Minnesota, Minneapolis, Minnesota;; ^3^ Center for Infectious Disease Emergency Response, Yong Loo Lin School of Medicine, National University of Singapore, Singapore

***Advocate***

*Cleaved leucocyte, shooting the moon*

*Stealthy virus, hijacking a duped cell*

*Fanged worm, questing for your blood*

*We see things hidden from the angels*

*We see them in*

*The man with the cantaloupe jaw*

*The woman sputtering out each brittle word*

*The boy pale as diluted sand*

*And we know: it is so very possible*

*To halt this march of death*

*Think of the truths you hold closest to your heart*

   *This*

*This*

    *This*

*This*

   *This*

*Who knows them*

*When you hide them in your room?*

*The cardinal argues for spring*

*For the delicacy of all we would destroy*

*Learn from the cardinal*

*Advocate*

       ∼ Chandy C. John

Karen A. Goraleski joined the American Society of Tropical Medicine and Hygiene (ASTMH) as its Chief Executive Officer (CEO) in 2010, and she stepped down as CEO in 2023 after a remarkably successful tenure with the Society. We offer here a short tribute to her outstanding service to the ASTMH and to the global health community.

Karen is a visionary, who in her 13-year tenure as CEO worked with ASTMH Presidents and the Board to transform the organization. Numbers tell part of the story… During Karen’s tenure as CEO, ASTMH experienced remarkable growth. In 2010, ASTMH had 2,721 members and 3,280 people attended the Annual Meeting; in 2023, ASTMH had 4,193 members and 4,572 people attended the Annual Meeting. But numbers are only part of this story.

During these 13 years, Karen, ASTMH Presidents, and the Board moved ASTMH forward in a number of key areas, including moving the Society to its current status as an independent organization with its own administrative structure. This critical decision and its implementation resulted in the Society being able to expand both staff and influence, as we moved to the Washington, DC area. Additionally, Karen worked with the Presidents and Board to support a growing staff group with exemplary skills and dedication; develop an Inclusion and Respect policy; create Board positions specifically for trainees; create a new subgroup, the ASTMH Committee on Global Health; significantly expand trainee travel awards, in particular, awards for those from low- and middle-income countries; create the Green Task Force to promote initiatives that combat climate change; create the first Society Medal named for a female Society Member, the Clara Southmayd Ludlow Medal; create the Communications Award to highlight the importance of science communication in all scientific work; work to address a gap in United States underrepresented minority membership and leadership; and substantially increase female and LMIC member leadership in the Society. After the untimely death of past ASTMH President Alan Magill, Karen helped spearhead a fund-raising effort in his honor, culminating in the Alan J. Magill Fellowship, funded by the Bill and Melinda Gates Foundation, awarded to a recipient from a low or lower middle-income country, and focusing on leadership development in tropical medicine and global health. Recipients since 2017 are proving to be leaders in their fields.

Karen was famous for her attention to the details of the Society, ranging from marketing to detailed notes for presidents at Board meetings, to big picture items which positioned ASTMH for success. These contributions, and many others, played major roles in the work and impact of the Society, but Karen’s greatest gift to the Society may have been her role in advancing ASTMH via advocacy for the work we do and the groups we work with. Our partnership with Venable LLC in advocating for the work of the Society and Society members has been key in getting the word out about what we do, why it matters to policymakers in the US and internationally, and why it should be supported. This was particularly notable in 2017, when ASTMH strongly advocated for the importance of the Fogarty International Center after a proposal to eliminate it. As part of a group of key stakeholders, ASTMH provided vital information to the public and policymakers on the work of the Fogarty International Center and why it is important for health and security in the United States and abroad, and support for the Fogarty International Center was maintained by the US government. Karen also supported an ASTMH Annual Meeting focus in 2017 on refugee and immigrant health, with a clinical pre-meeting course on migrant health, and a state-of-the-art interactive educational exhibit on refugee health, in collaboration with the CDC.

Karen’s advocacy work during her time at ASTMH involved working with multiple key global health partners, including service on the boards of the Global Health Council and the Campaign for Public Health; on the Steering Committee of the Global Health Technologies Coalition; and as an advisor to the Pan American Health Organization Malaria Champions Group. Advocacy for the work of ASTMH and partner organizations that work in global health is particularly important in the current climate, where powerful voices argue against scientific evidence as the basis for policy and funding in health in the United States and globally. We owe a tremendous debt to Karen for her pioneering work within the Society on advocacy which has led to our ongoing involvement in this very important aspect of our work.

The Society is profoundly grateful to Karen for all the work she did as CEO to make us a better, stronger, more inclusive, and higher impact organization. ASTMH President Daniel G. Bausch presided over a farewell for Karen at the 2023 Annual Meeting in Seattle, Washington. As part of the farewell, he asked past President Chandy C. John (2019) to read a poem he wrote as a tribute to Karen. The poem, reflecting Karen’s most passionate goal for the Society, was called, “Advocate”.

